# Simulation and Machine Learning Methods for Ion-Channel Structure Determination, Mechanistic Studies and Drug Design

**DOI:** 10.3389/fphar.2022.939555

**Published:** 2022-06-28

**Authors:** Zhengdan Zhu, Zhenfeng Deng, Qinrui Wang, Yuhang Wang, Duo Zhang, Ruihan Xu, Lvjun Guo, Han Wen

**Affiliations:** ^1^ Academy for Advanced Interdisciplinary Studies, Peking University, Beijing, China; ^2^ Beijing Institute of Big Data Research, Beijing, China; ^3^ DP Technology, Beijing, China; ^4^ School of Pharmaceutical Sciences, Peking University, Beijing, China; ^5^ National Engineering Research Center of Visual Technology, Peking University, Beijing, China

**Keywords:** ion channel, cryo-EM, machine learning, molecular dynamics, computer-aided drug design

## Abstract

Ion channels are expressed in almost all living cells, controlling the in-and-out communications, making them ideal drug targets, especially for central nervous system diseases. However, owing to their dynamic nature and the presence of a membrane environment, ion channels remain difficult targets for the past decades. Recent advancement in cryo-electron microscopy and computational methods has shed light on this issue. An explosion in high-resolution ion channel structures paved way for structure-based rational drug design and the state-of-the-art simulation and machine learning techniques dramatically improved the efficiency and effectiveness of computer-aided drug design. Here we present an overview of how simulation and machine learning-based methods fundamentally changed the ion channel-related drug design at different levels, as well as the emerging trends in the field.

## Introduction

**FIGURE 1:**
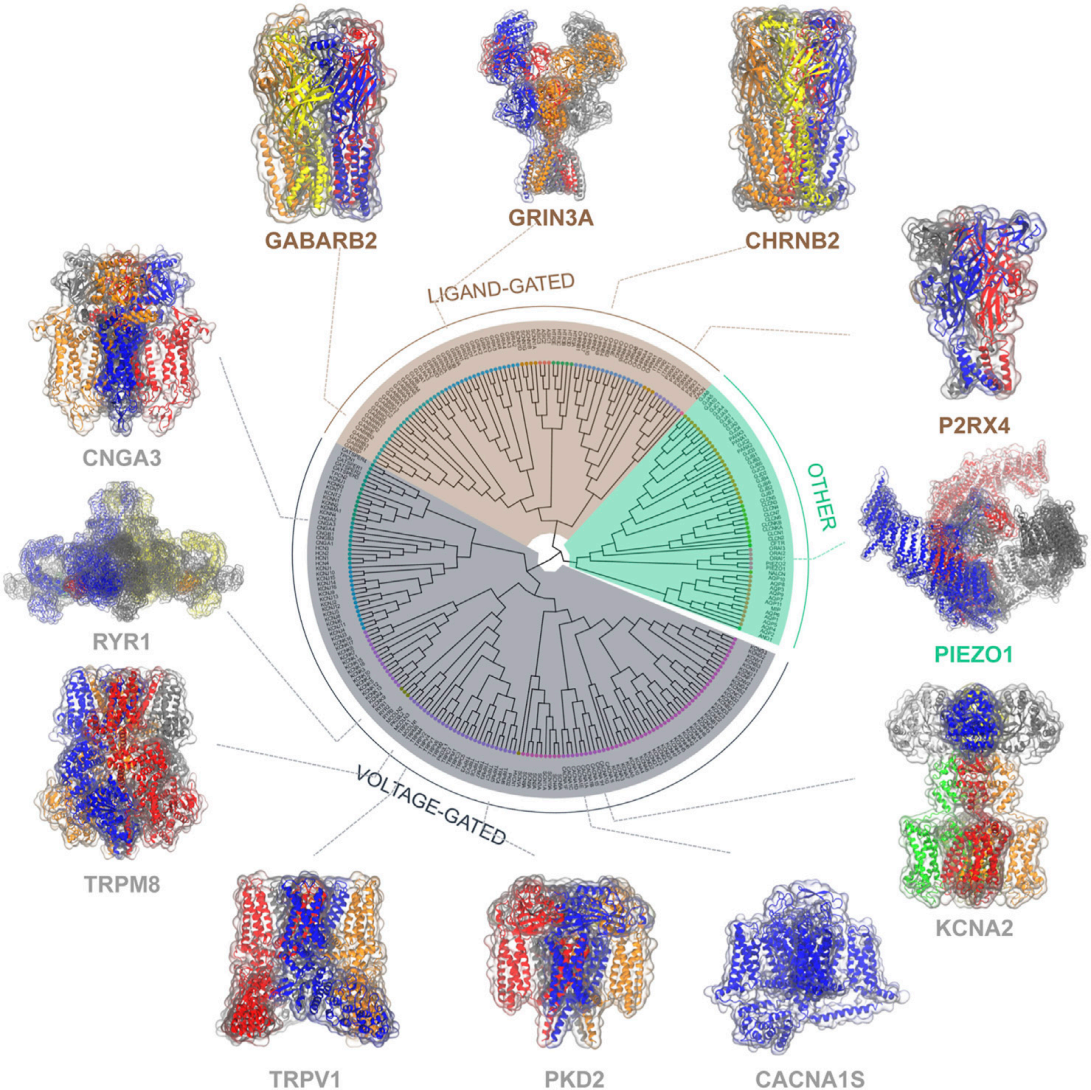
Classification of ion channels based on activation mechanisms and structural similarities. The evolutionary relationships among these ion channels are measured by the phylogenetic tree of ion channels (Alexander et al., 2019) with representative channel structures (center). Representative ion channels selected from each class are shown with their 3D molecular structures.

Ion channels are a group of pore-forming proteins located at the membrane of cells or intracellular organelles, controlling the flow of ions across the membranes. Such transportation of ions plays a critical role in the physiology of all living cells (Hille, 1978; Hille, 1986), thus the malfunction of ion channels leads to numerous diseases at the fundamental level. Although spanning a broad spectrum of families, ion channels can be roughly categorized into the voltage-gated and the ligand-gated based on activation mechanisms and structural similarities (Figure 1). Most voltage-gated ion channels contain four individual subunits or four repeats within one continuous polypeptide, each includes a similar six-transmembrane-helix core architecture, which can be further divided into a four-helix voltage sensor domain S1-S4 and the pore domain S5-S6 connected by the pore loop (Gulbis et al., 1999). The typical voltage-gated ion channels, mainly the sodium (Catterall, 2000), potassium (Wulff et al., 2009), and calcium channels (Catterall, 2011), are mostly depolarization activated and responsible for fast reactions upon voltage changes at the cell membrane, thereby playing a key part in electric signaling (Catterall, 2000; Sands et al., 2005; Catterall, 2010). While some members of the transient receptor potential (TRP) family, mainly facilitate their biological function via other stimuli including temperature, force, and chemical compounds. They serve as the vanguard of the sensory system (Clapham, 2003). As for ligand-gated ion channels, they are mostly activated by ligands like neurotransmitters and are crucial for nervous activities. There are three families in mammals: Cys-loop receptor ion channels (Sine and Engel, 2006), Glutamate receptor ion channels (Traynelis et al., 2010), and ATP gated ion channels (Schmid and Evans, 2019), they are typically composed of a transmembrane domain forming the pore and an extracellular domain carrying the ligand-binding sites. Besides, some other ion channels are beyond these two types, for example, the ubiquitously expressed trimeric Piezo 1/2 channels that respond to mechanical stimuli (Coste et al., 2010).

Due to the vital function of ion channels in physiology, mutations of ion channels were identified to be responsible for various diseases: inherited neuronal diseases like epilepsy (Escayg et al., 2000), cardiac diseases like Long-QT syndrome (Keating, 1996), muscle diseases like Hyperkalemic periodic paralysis (Brugnoni et al., 2022) and many others (Ackerman and Clapham, 1997). Also, the blockage of ion channels can directly regulate sensory perception such as pain (Tominaga and Caterina, 2004; Bourinet et al., 2014; Bennett et al., 2019). Consequently, ion channels are among the most pursued drug targets in the past decades (Bagal et al., 2013) and several drugs were developed against a wide range of diseases (Clare, 2010). To be specific, for the most related central nervous system (CNS) diseases: Diazepam, the famous anxiolytic launched in 1963 and the best-seller in the United States between 1968 and 1982 (Calcaterra and Barrow, 2014), was identified as a positive allosteric modulator of the gamma-aminobutyric acid (GABA) type-A receptors in 1977 (Braestrup and Squires, 1977; Mohler and Okada, 1977). A detoured but successful development of ketamine, the *N*-methyl-D-aspartate (NMDA) receptor antagonist (Alessandri et al., 1989; Zhang et al., 2021) into an anti-depressant drug is even considered a sign of a renaissance in the psychiatric drug industry (Daly et al., 2018; Reardon, 2018). Recent studies also revealed more potential applications of channel modulators in Parkinson’s (Daniel et al., 2021) and Alzheimer’s diseases (Tan et al., 2012; Gonzales and Sumien, 2017; Lu et al., 2017). Additionally, the prevalent expression makes ion channels adequate targets for other diseases as well. Varenicline is derived from the modification of (-)-cytisine, an alkaloid that could be found in nature (Coe et al., 2005). As a partial agonist of nicotinic receptors with the beta 2 subunit (Papke and Heinemann, 1994), Chantix (varenicline tartrate) was launched by Pfizer in 2006 for smoking cessation (Coe et al., 2005) and is still one of the best-selling drugs in the world with annual sales of 919 million dollars in the year of 2020 (McGrath et al., 2010; Njardarson et al., 2020). In addition, cystic fibrosis, a heritable disease, is caused by a defect in the ATP-activated chloride channel, cystic fibrosis transmembrane conductance regulator (CFTR) which is involved in water flow control during the production of sweat, digestive fluids, and mucus (Bobadilla et al., 2002). The latest treatment for cystic fibrosis, Trikafta, was developed by Vertex Pharmaceuticals and hit the market in 2019, and was considered at the top of 2019's new approvals by expected revenue (Mullard, 2020). Another case for diabetes treatment is the blocking of ATP-sensitive potassium channel KATP by Glipizide leads to depolarization of beta cells, resulting in the opening of voltage-gated calcium channels which encourages insulin release (Shuman, 1983). Ion channels were also reported to be potential anti-cancer targets. Their role in proliferation, migration, and metastasis of cancer was well-explored (Litan and Langhans, 2015) and the over-expression of TRPA1 and TRPV1 channels was observed in several tumors suitable for topical administration, allowing for a safe and efficient therapy (Kiss et al., 2020).

Despite the vast opportunities, the discovery of drugs targeting ion channels faces unique obstacles. To begin with, the development of biological assays for ion channels is usually challenging, and the low expression rates further hindered the high-throughput screening. Ever since Hodgkin and Huxley firstly described the movement of ions in nerve cells of squid axons during an action potential (Hodgkin and Huxley, 1952), electrophysiology has been used to study the function of ion channels. Yet, such methods require considerable expertise and are labor-intensive and time-consuming, therefore not realistic for large-scale screening, even with the great advancement in automated patch clamp devices (Dunlop et al., 2008; Kodandaramaiah et al., 2012). Another major stumbling block is ligand selectivity because channels from the same or close families usually share great similarities in sequence and structure yet bear diverse functions. Lead compounds, especially channel blockers, often suffer from side effects due to poor selectivity (Vandenberg et al., 2017; Flood et al., 2019). As the “low-hanging fruit” is becoming rare in modern drug discovery, cases like carbamazepine, a pan-ion-channel inhibitor with broad pharmacological properties (Bagal et al., 2013), may be very rare and not welcomed nowadays, for which mechanism studies came several decades later than discovery (Schindler and Hafliger, 1954; Grant and Faulds, 1992).

Because of the aforementioned problems, the rational design of drugs is critical for the efficiency and efficacy of ion channel drug discovery. Such designs require precise structural information, which is largely missing until the rise of single-particle cryo-electron microscopy (cryo-EM) (Li et al., 2013; Liao et al., 2013). Starting from the high-resolution structures, computer-aided drug design (CADD) can be applied to perform large scale virtual screenings before the costly wet-lab experiments to greatly narrow down the size of the library to an affordable range (Lyne, 2002); molecular dynamics (MD) simulations can be performed to further model the structures and study the mechanism at atomistic details (Maffeo et al., 2012). On the other hand, recent advancement in machine learning begins to demonstrate their power in cryo-EM and drug development. Towards accelerating the ion channel drug discovery, in this review, we will provide a broad overview of the current machine-learning and simulation-based techniques in structural biology and drug design, and discuss how they can be applied to ion channel research.

## Computational Methods for Single-Particle Cryo-EM

While being essential for mechanistic studies and rational drug design, molecular structures for ion channels are more difficult to purify and crystallize than soluble proteins, mainly due to the necessity and difficulty of preserving the membrane-like environment. Detergents, amphipols, and nanodiscs have been commonly used to extract membrane proteins such as ion channels, and also serve as substitutes for the local membrane to stabilize the transmembrane domains (Zampieri et al., 2021). In recent years, the single-particle cryo-EM technique has rapidly evolved as a powerful method for structure determination for various ion channels (Martin et al., 2017; Basak et al., 2019; Dang et al., 2019; Masiulis et al., 2019; Zhao et al., 2019; Wang Q. et al., 2020; Sun and MacKinnon, 2020; Lin et al., 2021; Song et al., 2021; Yu et al., 2021). Cryo-EM eliminates the need for protein crystallization and is capable of resolving multiple protein conformations within a single cryo-EM dataset (Frank and Ourmazd, 2016; Frank, 2017; Cheng, 2018; Wu and Lander, 2020). The structural resolution of cryo-EM experiments has also been improved over the years, with the highest resolution reaching ∼1.2 Å (Nakane et al., 2020; Yip et al., 2020). High-resolution cryo-EM enables its use in target identification and lead-optimization for developing drugs targeting ion channels.

**FIGURE 2:**
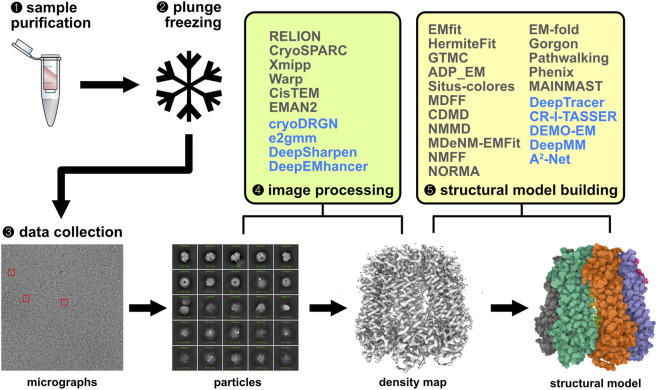
Singe-particle cryo-EM workflow and relevant computational methods. Traditional methods for image processing and structural model building are shown in grey and ML-based methods in blue. Electron micrographs and particle images were visualized using cryoSPARC (Punjani et al., 2017). The density map and structural model were generated using Mol* viewer (Sehnal et al., 2021).

A typical single-particle cryo-EM experiment includes sample purification, vitrification via plunge freezing, electron microscopy data collection, image processing, and structural model building (Figure 2) (Lyumkis, 2019; Wu and Lander, 2020). We will focus on the last two steps in this review, which rely heavily on computational techniques.

### Cryo-EM Image Analysis

The goal of cryo-EM image analysis is to reconstruct the three-dimensional (3D) structure of the target proteins represented as statistically estimated density maps. A large collection of two-dimensional (2D) projection maps are subjected to image preprocessing, particle picking, 2D image classifications, 3D classifications, and 3D reconstructions (Singer and Sigworth, 2021). Many software programs are available for building cryo-EM density maps from 2D projection maps, such as RELION (Scheres, 2012; Kimanius et al., 2021), cryoSPARC (Punjani et al., 2017), Xmipp (Sorzano et al., 2004), Warp (Tegunov and Cramer, 2019), CisTEM (Grant et al., 2018), EMAN2 (Tang et al., 2007). Although these programs are sufficient for most cryo-EM experiments, their output density maps are mostly limited to a few representative classes of particles in the projection map dataset. Recent machine-learning-based methods focus on characterizing the continuous distribution of particle distributions within a cryo-EM experiment, e.g., cryoDRGN (Zhong et al., 2020; Zhong ED. et al., 2021; Zhong E. D. et al., 2021) and e2gmm (Chen and Ludtke, 2021). CryoDRGN is a method for heterogeneous cryo-EM reconstruction based on deep neural networks. It is powerful and general for analyzing structural heterogeneity in macromolecular complexes of various sizes and degrees of heterogeneity. The cryoDRGN model consists of two neural networks structured in an image-encoder-volume-decoder architecture with a continuous latent variable representation for describing the sample heterogeneity. Although the initial version of cryoDRGN (Zhong et al., 2020) can optimize image poses together with image reconstruction/classification, for better efficiency and accuracy, the pose estimation component was dropped and pre-computed image poses estimated from an upstream homogeneous reconstruction were used (Zhong ED. et al., 2021). CryoDRGN2 combines a traditional cryo-EM image pose search algorithm with deep neural networks, which eliminates the need for pre-computed image poses (Zhong E. D. et al., 2021). Compared with the initial version of cryoDRGN, both the speed and accuracy are increased significantly in pose estimation. The e2gmm algorithm can also find the structural variability of target macromolecules captured by cryo-EM experiments. In this method, each structural domain is modeled by a 3-dimensional (3D) Gaussian function, parameterized by its center coordinates, amplitude, and width. Autoencoder-like neural network architecture is used to generate a latent vector representation of each projection image through an encoder. Then the decoder component converts each latent vector into a set of 3D Gaussian function parameters which represents a Gaussian mixture model (GMM). This GMM representation is converted back to a 2D projection image through a projection operation. In e2gmm, training was first done for the decoder only, then for the encoder and decoder together. Unlike the standard autoencoder, the input to the encoder is not the projection image, but the gradient of loss function score with respect to the GMM parameters (Chen and Ludtke, 2021). The projection orientation of each image must be given as inputs which can be refined later once the network has been trained. The conformational variability of the cryo-EM dataset can be visualized through dimension reduction of the 4D latent space based on the latent vectors from the encoder. The cryoDRGN and e2gmm algorithms are similar in terms of the use of latent vectors for characterizing the structure variability, although latent vectors are obtained differently.

Reconstructed cryo-EM density maps usually suffer from loss of contrast at high resolution due to factors such as specimen movement, radiation damage, particle flexibility/heterogeneity, and deficiencies in extracting/averaging micrograph signals in the reconstruction algorithms (Rosenthal and Henderson, 2003). Algorithms, known as sharpening, have been designed to reduce this type of contrast loss. A commonly used sharpening method is based on global B-factor correction (Rosenthal and Henderson, 2003). Other methods, such as LocScale (Jakobi et al., 2017), LocalDeblur (Ramirez-Aportela et al., 2020), and LocSpiral (Kaur et al., 2021), improved upon the previous method by considering local variations in B-factors. Instead of explicitly performing sharpening calculations, deep-learning-based methods can implicitly encode the sharpening transformation. One example is DeepSharpen (Zehni et al., 2020), which uses a Convolutional Neural Network (CNN) trained on ∼15,000 pairs of low and high-resolution synthetic cryo-EM density maps. The benefit of this approach is that sharpening on a new density map only requires inference through trained CNN parameters which can be very fast. Deep cryo-EM Map Enhancer (DeepEMhancer) is another deep-learning-based sharpening method (Sanchez-Garcia et al., 2021). It uses a 3D U-net architecture (Ronneberger et al., 2015) to learn the local sharpening effect of the LocScale algorithm by training on paired map datasets from the Electron Microscopy Data Bank (EMDB). Each map pair consists of an experimental density map and a post-processed map using LocScale. DeepSharpen and DeepEMhancer share similar deep learning designs, although their training strategies and training targets differ. More realistic and better-labeled training datasets can further improve these supervised-learning-based methods.

### Cryo-EM Structural Model Building

In the final step of cryo-EM experiments, 3D atomic structural models are built based on the reconstructed 3D density maps. The goal is to search for the most probable 3D atomic structure that can best interpret the reconstructed density map. Traditionally, these atomic structural models are built semi-automatically using software like Coot (Casañal et al., 2020) and Phenix (Liebschner et al., 2019). These types of methods require the users to have extensive knowledge of the biochemical compositions of the target biological entities. More automated methods can be roughly divided into three categories. The first class of automated methods performs rigid-body fitting of initial structures into the density maps. Examples of such methods include EMfit (Rossmann et al., 2001), HermiteFit (Derevyanko and Grudinin, 2014), GTMC (Wu et al., 2003), ADP_EM (Garzón et al., 2007), and Situs-colores (Chacón and Wriggers, 2002). These methods solve the problem of placing an atomic structure model into a target cryo-EM density map as a rigid body and they differ in their specific implementation of translational/rotational search. Most of these methods are developed for low-resolution maps before the so-called “resolution revolution” during the development of cryo-EM (Crowther, 2016; Frank, 2017; Alnabati and Kihara, 2019).

As the average resolution of cryo-EM increased, the second class of automated methods emerged which allows flexible fitting of initial structures into density maps. One type of flexible fitting method combines molecular dynamics (MD) simulations and the experimental density maps to automatically fit some initial structural models into density maps. Examples of such methods include molecular dynamics flexible fitting (MDFF) and correlation-driven molecular dynamics (CDMD). In MDFF (Trabuco et al., 2008; Chan et al., 2011; Singharoy et al., 2016), an additional potential energy term derived from the density map was added to the simulation force field. The derivative of this potential generates forces that drive the initial structural model into the target density map. CDMD acts similarly (Igaev et al., 2019). Instead of directly adding a density-map-derived potential energy term, the cross-correlation coefficient (ccc) between the intermediate structures and the target density map is calculated. Forces derived from ccc drive the initial structure into the density map. Although both methods are equivalent, CDMD is more sensitive to the quality of initial structures and noises in the density maps compared to MDFF in practice. ISOLDE is a hybrid method that combines user interactions and MDFF to achieve a semi-automated structure modeling (Croll, 2018). The same hybrid modeling can also be performed using interactive MDFF (iMDFF) (McGreevy et al., 2016). NMMD is another MD-based cryo-EM structural fitting method (Vuillemot et al., 2022). Compared to CDMD and MDFF, NMMD combines normal mode analysis (NMA) and MD to perform global structural fitting with NMA and local structural fitting with MD. Other NMA-based flexible fitting methods include MDeNM-EMFit (Costa et al., 2020), NMFF (Tama et al., 2004a; b), NORMA (Suhre et al., 2006), and a coarse-grained fitting method with a modified elastic network model (Zheng, 2011). Besides MD, Monte Carlo (MC) simulations together with MD can also be used to fit structures into density maps (Topf et al., 2008).

Another approach to building cryo-EM structures is *de novo* modeling, which doesn’t require any initial structural models. This class of methods includes RosettaES (Frenz et al., 2017), EM-fold (Lindert et al., 2009; Lindert et al., 2012a; Lindert et al., 2012b), Gorgon (Baker et al., 2011), Pathwalking (Baker et al., 2012; Chen et al., 2016), Phenix (Terwilliger et al., 2018; Terwilliger et al., 2020), MAINMAST (Terashi and Kihara, 2018b; a), etc. (see the recent review (Alnabati and Kihara, 2019)). More recently, deep-learning-based *de novo* cryo-EM structural model building has been gaining much ground, such as DeepTracer (Pfab et al., 2021), CR-I-TASSER (Zhang et al., 2022), DeepMM (He and Huang, 2021), and A^2^-Net (Xu et al., 2019). DeepTracer (Pfab et al., 2021) uses four U-Net-based neural networks to extract structural information from the input density amp. Each network can classify each density map voxel into a certain category. The Atoms U-Net predicts four classes: C-alpha, nitrogen, carbon (non-C-alpha), and non-atom. The Backbone U-Net predicts three classes: backbone, side chain, and neither. The Secondary Structure U-Net predicts four classes: loop, helix, sheet, and none of these. The Amino Acid Type U-Net predicts 21 classes: 20 types of amino acids and non-protein density. Then, protein backbone tracing is done in three steps: identifying protein chains based on Backbone U-Net outputs, estimating C-alpha atom coordinates, and connecting C-alpha atoms into chains using a modified traveling salesman algorithm. The assignment of amino acid residue types to each C-alpha atom was done through a customized sequence alignment algorithm based on the U-Net-predicted and input protein sequences. The all-atom backbone structure is subsequently reconstructed based on information from the Atoms U-Net predictions and some assumptions on the peptide bond geometries. One of the major limitations of DeepTracer is its inability to predict the side-chain conformations, which is delegated to an external program called SCWRL4 (Krivov et al., 2009). CR-I-TASSER (cryo-EM iterative threading assembly refinement) is a hybrid method that uses information from both homology modeling and density-map-based *de novo* C-alpha tracing (Zhang et al., 2022). Like DeepTracer, C-alpha atom positions are predicted based on density maps using machine learning techniques, which is a deep convolutional network (3D-CNN) in this case. Then, CR-I-TASSER solves the problem of C-alpha tracing using a local meta-threading server (LOMETS). After that, the atom structure models are built using the iterative threading assembly refinement method (I-TASSER) driven by model-map correlation under deep-learning boosted template restraints. The novelty of this method is the C-alpha position prediction using 3D-CNN and the use of C-alpha locations for better template reselection in LOMETS. CR-I-TASSER shows good performance in terms of TM scores compared to other methods such as MAINMAST and MDFF. However, its accuracy is limited for low-resolution cryo-EM data, and its C-alpha tracing method is also problematic in long-loop/tail or disordered regions. Another major limitation of CR-I-TASSER is that it can only handle single-chain proteins, thus the density maps have to be manually segmented first. DeepMM takes a workflow design similar to DeepTracer (He and Huang, 2021). Based on cryo-EM density maps, it uses one Densely Connected Convolutional Network (DenseNet) to predict the main chain (backbone N, C, and C-alpha atoms) and C-alpha probabilities at each density map voxel. Using the MAINMAST method, the main-chain paths are determined based on the main-chain probability map. Then, another DenseNet is used to predict the residue identities and secondary structure types for each main chain point. Smith-Waterman dynamic programming is used to align the target sequence to the main-chain paths. Finally, all-atom structure models are based on the top-10 C-alpha models using the ctrip program (Xiang and Honig, 2001; Petrey et al., 2003) and refined by energy minimization using Amber (Case et al., 2005). Like CR-I-TASSER, DeepMM can only handle building single protein chains and segmentation tools, such as Segger (Pintilie et al., 2010) are needed to preprocess multi-chain density maps into individual chain map segments. Compared to DeepTracer, DeepMM’s main feature is the ability to build full-length protein structures from high-resolution EM maps, while DeepTracer only builds backbone segments in high-resolution map regions. All these methods only apply machine learning techniques to convert cryo-EM maps into basic protein structural models without side chains. A2-Net (Xu et al., 2019) is another *de novo* structure building method based on a deep Convolutional Neural Network (Srivastava et al., 2015). Like the methods mentioned above, it contains neural networks for amino acid residue identity detection. The main difference is that A2-net also contains a poseNet based on the 3D stacked hourglass network (Newell et al., 2016) for estimating the coordinates of each residue (side chain included). Then, these residues are connected to form the target protein using a Monte Carlo tree search algorithm. In the advent of accurate protein structure prediction methods such as AlphaFold2 (Jumper et al., 2021) and RoseTTAFold (Baek et al., 2021), new possibilities are brought to cryo-EM structure building. For example, ChimeraX added a new feature to allow users to start cryo-EM structure modeling from AlphaFold predictions^1^. Phenix also added a new workflow^2^ (Terwilliger et al., 2022), to iteratively refine cryo-EM structural models by integrating AlphaFold predictions using ColabFold (Mirdita et al., 2022). Recently, Yan and Shen reported that AlphaFold2-predicted structure could help model the density map regions with moderate resolutions in voltage-gated sodium channel Na_v_1.7 (Huang et al., 2022). With the rapid progress in the field of machine learning, more accurate and efficient *de novo* cryo-EM structure modeling methods are expected in the near future.

### Evaluation of the Cryo-EM Structural Model Quality

Evaluation of the quality of the density-map-derived molecular structural models is yet another challenge. Global metrics (such as map-model cross-correlation coefficient and FSC curve) between the experimental density map and a simulated map converted from the structural model are often insensitive to local mis-fittings (Hryc et al., 2017). Moreover, these metrics usually do not account for densities not modeled by the atomic structural models such as lipids and detergents, and masks are usually needed to access local features (Pintilie et al., 2016). EMRinger and Z-scores are metrics defined based on how well the structure models interpret the experimental density maps. EMRinger score is based on the expectation that the map density values near the beta-carbon atoms should peak at certain angles when rotating the side-chain chi-1 dihedral angle (Barad et al., 2015). Although the EMRinger score correlates well with the protein backbone modeling quality and map density quality near the beta-carbons, it is suspectable to noise and lacks assessment of the rest of the side chains. Z-score has been proposed to quantify the density map quality at the secondary structure element level and side-chain level (Pintilie and Chiu, 2018). Its definition is based on the cross-correlation coefficients between the experimental and simulated maps of the secondary structure elements or side chains with and without some geometric displacements (Pintilie and Chiu, 2018). To make even finer-scale assessments, Q-score is established to quantify the resolvability of individual atoms in cryo-EM maps (Pintilie et al., 2020). The basic idea of the Q-score is to measure the cross-correlation coefficient between experimental density map values for individual atoms and ideal densities represented by a three-dimensional (3D) Gaussian-like function centered at each atom in the structure model (Pintilie et al., 2020). FSC-Q is another method that can also assess structure modeling quality on a per-atom basis (Ramírez-Aportela et al., 2021). FSC-Q calculates the difference between two local resolution maps calculated using the blocres program. The first local resolution map is calculated using the full density map and the simulated map based on the structure model, while the second is calculated using the half maps. Then the FSC-Q difference map can be projected onto atomic models to obtain an FSC-Q score for each atom. Alternatively, the FSC-Q map can be normalized by dividing by the local resolution map based on the half maps for better cross-comparison between experimental density maps of different resolutions.

### Challenges in Cryo-EM Structure Determination for Ion Channels

Ion channels naturally reside in biological membranes which means preserving the amphiphilic local environment for the channel transmembrane domains is essential during cryo-EM sample preparation. Techniques such as nanodiscs can serve as good replacement for the membrane (Zampieri et al., 2021). However, the disordered nature of these membrane memetics also add difficulties to the cryo-EM structural analysis. In cryo-EM image analysis, algorithms such as non-uniform refinement can reduce the negative impact of the disordered detergent/lipid molecules (Punjani et al., 2020). In addition, certain ion channels, such as mechanosensitive channels, have flexible transmembrane domains and often require specific protein-lipid interactions for structural stability (Kefauver et al., 2020). Preserving their local membrane environment can be very difficult. For example, it has been reported that mechanosensitive channel YnaI’s transmembrane domain couldn’t be resolved using cryo-EM with membrane-active polymer SMA2000 except for core helices TM4 and TM5 (Catalano et al., 2021). Computational techniques such as normal model analysis and molecular dynamics can offer additional insights into these protein-lipid interactions required for the structural stability and functions of mechanosensitive channels (Jojoa-Cruz et al., 2018; Argudo et al., 2019). In addition, the application of toxins, truncation of flexible regions and mutations are often introduced in cryo-EM studies of ion channels (Jiang D. et al., 2021). Although sometimes intended by the researchers for their specific projects, these conditions have also been used to artificially stabilize ion channels to capture a certain conformation during structure determination (Rook et al., 2021). Computational techniques can reverse these effects if needed and can also be used to evaluate the impact of such conditions (Gupta and Vadde, 2021; Saikia et al., 2021).

## Computer-Aided Drug Design Approaches Targeting Ion Channels

In the glorious history of the era driven by occasions and classic synthetic chemistry, numerous ion-channel modulators were discovered. For example, the series of “-caine” analogues was inspired and chemically modified from cocaine, an alkaloid derived from plants (Plowman, 1982), including benzocaine, amylocaine, procaine, procainamide, and lignocaine (lidocaine), which showed potency as sodium channel inhibitors and were approved for medical use as anesthetics and/or antiarrhythmics (Cox, 2015). Typical cases also include the previously mentioned varenicline and carbamazepine, and maybe even ethanol and neurotoxic peptides in venoms. Ethanol was proved to be able to interact with various post-synaptic ion channel receptors (Howard et al., 2011; Murail et al., 2012; Zhang et al., 2016), while neurotoxic peptides in venoms were sometimes regarded as good starting points for modification as modulators targeting ion channels such as voltage-gated sodium, potassium, and calcium channels as well as some ligand-gated channels (Clark et al., 2010; Dutertre and Lewis, 2010; Bagal et al., 2013; Wulff et al., 2019).

**FIGURE 3:**
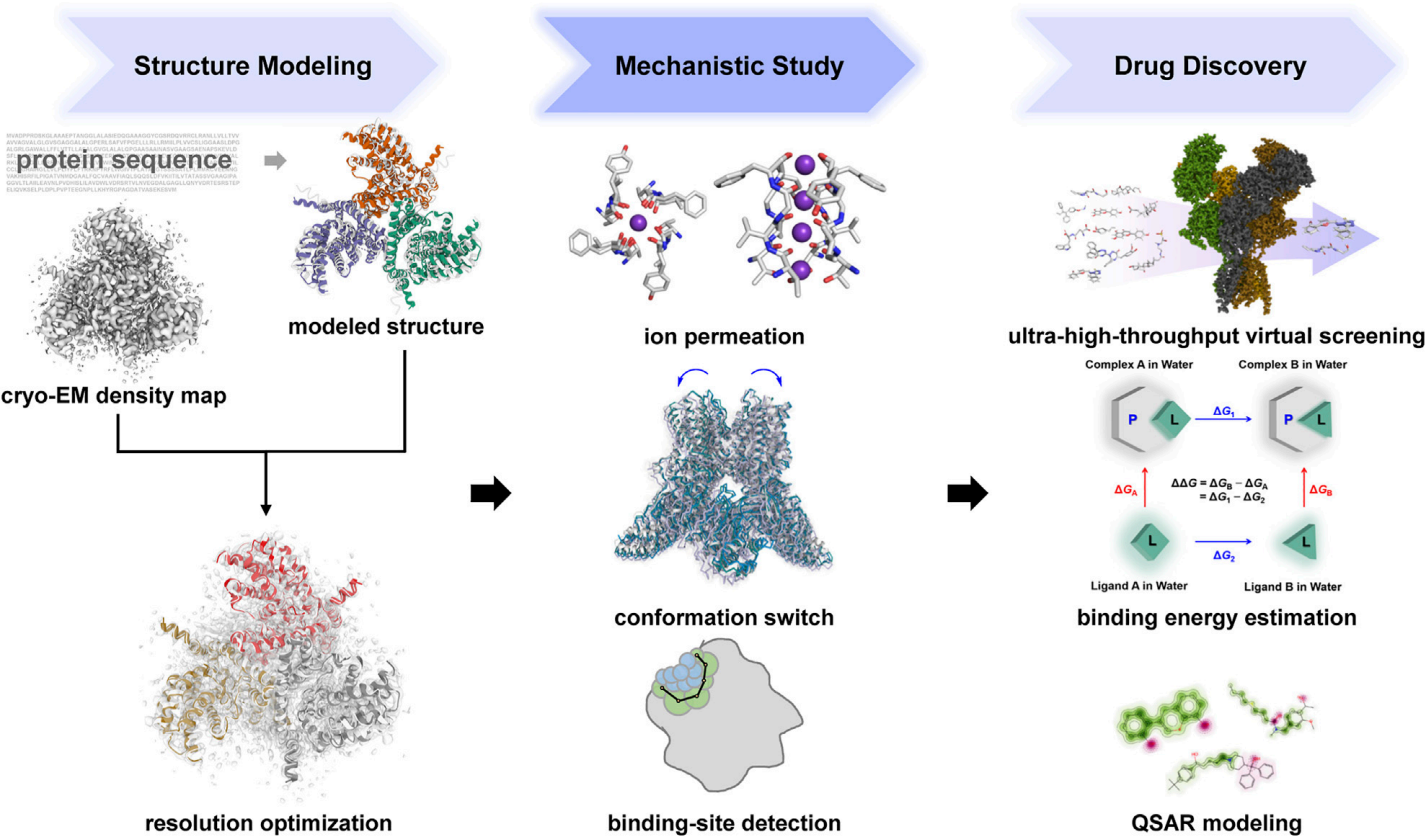
Computational approaches in the structure modeling, mechanistic study and drug discovery of ion channels. Several subplots were collected using Hermite (https://hermite.dp.tech), Mol* viewer (Sehnal et al., 2021), PyMOL (https://github.com/schrodinger/pymol-open-source) and the Pred-hERG web-server (Braga et al., 2015).

In recent years, under the concept of rational drug design, considering the features of ligands, the structures of receptors, and the motions and dynamics of the system becomes more and more important. Accordingly, numerous computational methods and machine-learning algorithms have been developed (Figure 3).

### Ligand-Based Approaches

Benefit from these data collected in the early era, ligand-based approaches could be applied to build relatively reliable models to describe the relationship between modulators and the corresponding ion channels, especially when the experimentally-resolved protein structures are absent. One of the typical cases should be the development of ligand-based models for the prediction of human ether-a-go-go related gene hERG potassium ion channel blockage, which is believed to be important in avoiding sudden cardiac death considering its relationship with drug-induced long QT syndrome. In the past few years, a considerable amount of associated data was collected (Konda et al., 2019) and numerous quantitative structure-activity relationship (QSAR) or classification models (Cavalli et al., 2002; Ekins et al., 2002; Roche et al., 2002; Keseru, 2003; Nisius and Goller, 2009; Braga et al., 2015; Siramshetty et al., 2018; Konda et al., 2019) were built for hEGR blockage prediction based on the traditional regression models such as partial least squares, machine learning algorithms including support vector machines (SVM), random forest (RF), gradient boosting machine (GBM) or neural network models. These models have been systematically discussed in the previous reviews (Aronov, 2005; Taboureau and Jorgensen, 2011; Villoutreix and Taboureau, 2015; Vandenberg et al., 2017; Menke et al., 2021).

Actually, besides hERG, the application of the mentioned models is also a common strategy in ligand-based drug design of ion channel modulators when facing abundant research data. For example, in the study of structural-activity relationships of Na_v_1.4 blockers, both traditional two-dimensional and advanced three-dimensional QSAR models were applied by Carrieri et al. to reveal the structural characteristics of bioactive molecules (Carrieri et al., 2009). Further design of sodium channel blockers was thus inspired. Consequently, a series of tocainide analogues were designed and one of them was verified to have a favorable pharmacodynamic profile and was proposed to be a valid Na_v_1.4 blocker (Muraglia et al., 2014). Based on the data collected from ChEMBL, BindingDB, and in-house databases, various machine learning-based QSAR models were built by Kristam et al. to predict blockers of the voltage-gated sodium ion channel Na_v_1.5, with the balanced accuracy of 0.88 (at the threshold of 1 μM) and predicted *R* ^2^ of 0.71 (RMSE = 0.73 for pIC50) for the classification and regression models, respectively (Khalifa et al., 2020). Similarly, Huang and Xie et al. trained and filtered a classification model based on the data from ChEMBL and BindingDB for the discovery of Na_v_1.7 blockers (Kong et al., 2020). The Grammar Variational Autoencoder, the trained classification model, and simulated annealing were combined to conduct the molecular optimization and an active compound was found and identified experimentally (Kong et al., 2020). Li et al. studied the pharmacophore hypothesis of ligands that bind at the benzodiazepine site of GABA_A_ receptors based on ligand-based pharmacophore, 3D-QSAR analysis, and Bayesian models, which might provide useful viewpoints in the discovery of GABA_A_ modulators (Yang Y. et al., 2013). Because of the potentially limited chemical space the pore-blockers could adapt, approaches based on the three-dimensional similarity between the ligands and reference molecules that take the spatial molecular shape and electrostatic features into consideration (Cleves et al., 2019; Jiang Z. et al., 2021) are possibly another practical ligand-based method in discovering ion channel modulators. Additionally, Bahar et al. proposed a probabilistic matrix factorization (PMF) based scheme to predict unknown drug-target interactions, which focused on the phenotypic similarity of the drugs by grouping the drugs according to the corresponding therapeutic effects (Cobanoglu et al., 2013). The obtained model showed good performance when applied to large data sets of ion-channel-drug pairs (Cobanoglu et al., 2013).

### Structure-Based Approaches

In terms of the structural-based approaches, recent computational and machine-learning technologies have empowered the exploration of ion channel modulators mainly in three aspects, viz., structures with higher quality, binding affinity prediction with higher accuracy, and virtual screening with higher throughput (Figure 3).

The recent rapid development of protein structure modeling, as well as structural biology, possibly enables the rational design and exploration of ion channel modulators with atomic-resolution structures. Taking again the case of hERG as an example, in the past, owing to the lack of solved hERG channel structure, structural-based exploration mainly relies on structures from homology modeling. With the modeled structure and docked model, Vaz et al. illustrated that the π-stacking formed between the blockers and Phe656 in the protein as well as that of the cation-π interaction involving Tyr652 might be the potential key elements of the pharmacophore (Pearlstein et al., 2003). Similar conclusions associated with the important character of Phe656 were also drawn by the work of Åqvist et, al., which explored the binding of potential hERG blockers using docking and molecular dynamics combined with the linear interaction energy method to evaluate the binding affinities of the potential blockers (Osterberg and Aqvist, 2005). However, numerous experimental ion channel structures have been determined with the cryo-EM technique nowadays, including the human hERG structures that were resolved in 2017 (Wang and MacKinnon, 2017), which might provide a significant cornerstone of the structure-based prediction. Beneficial from the high-resolution structures of the TRPM8 channel in various states (Yin et al., 2018; Diver et al., 2019; Yin et al., 2019), Yang et al. conducted a rational design of the TRPM8 channel and developed a modality-specific inhibitor DeC-1.2, which showed the potential as a novel analgesic against oxaliplatin-induced neuropathic pain (Aierken et al., 2021). Additionally, various protein-folding and modeling tools were also developed (Baek et al., 2021; Jumper et al., 2021), which provide an alternative way for protein structure modeling besides the classic homology modeling approach (Huang et al., 2022), and may also contribute as a start point for the construction of single-particle cryo-EM structures (Figure 3). The application of molecular dynamics may be another powerful approach to structural exploration and refinement (Hollingsworth and Dror, 2018; Carnevale et al., 2021). For example, following a multistep protocol, Noskov et al. modeled the open, closed and open-inactive states of the hERG channel to explore the molecular mechanisms behind the state-dependent binding of potential hERG blockers (Durdagi et al., 2012). The missing elements in the homology model were complemented with Rosetta, following all-atom molecular dynamics simulation to refine the obtained structure. The Poisson-Boltzmann calculations conducted based on the ligand-channel complex obtained by the Glide induced-fit docking (IFD) protocol showed a good correlation with the experimental results (Durdagi et al., 2012). Also, starting from the modeled structure, Barakat et al. performed the structural refinement of the Na_v_1.5 ion channel based on a long-time molecular dynamics simulation of 680 ns in the lipid membrane bilayer (Ahmed et al., 2017). The binding affinities between the reported molecules and the obtained model of the channel were evaluated with AMBER-MM/GBSA. The results demonstrated a good computational-experimental correlation (Pearson coefficient = 0.7) to distinguishing drugs that block or not block the Na_v_1.5 channel (Ahmed et al., 2017).

Binding affinities of the ion channel modulators are usually evaluated after the construction of reliable modulator-channel complexes. As discussed above, numerous algorithms are available in the historical explorations for binding affinity prediction, including LIE, MM/PBSA, and MM/GBSA, and showed relatively satisfactory results (Osterberg and Aqvist, 2005; Durdagi et al., 2012; Ahmed et al., 2017). Nevertheless, to derive the theoretically more accurate results, the more rigorous defined alchemical methods such as free energy perturbation (FEP) could also be applied to access the binding potency of the channel modulators (Limongelli, 2020). Based on the extensive search for the isoflurane binding sites in the proton gated Gloeobacter violaceus ligand-gated ion channel (GLIC) in their previous study (Brannigan et al., 2010), Klein and Brannigan et al. performed free energy perturbations to evaluate the binding affinities of two anesthetics against GLIC, viz., isoflurane and propofol (LeBard et al., 2012). The evaluated affinities demonstrated good correlations with the previous electrophysiology data (LeBard et al., 2012). In the work of Rempe and Ren et al., FEP was used to determine accurately the thermodynamic stabilities of ion configurations for the potassium channel of *streptomyces* A (KcsA), by comparing the relative free energy difference between various configurations (Jing et al., 2021). The computation results showed good agreement with evidence derived from crystal structures, ion binding experiments, and mutagenesis experiments (Jing et al., 2021). Moreover, machine learning techniques could be introduced in large-scale screening considering the large computational cost of alchemical methods. Bhat et al. showed that it is possible and acceptable to perform more than 5000 FEP calculations in one lead optimization task with the acceleration of active learning (Konze et al., 2019). System-specific scoring functions might provide another efficient way for discovering ion channel modulators. These scoring functions could be constructed either with deep learning or machine learning methods alone or combined with physical-based models (Ain et al., 2015; Wang et al., 2019; Guedes et al., 2021). Numerous studies have demonstrated that the application of target-specific scoring functions outperforms the universal scoring functions on the validation set of DUD-E, which usually covers the major druggable target classes and ion channels are also included (Ain et al., 2015; Wojcikowski et al., 2017; Wang et al., 2019; Guedes et al., 2021).

The development of ultra-high-throughput virtual screening might also be a chance to accelerate the discovery of ion channel modulators. Though various experimental techniques including ion-flux or fluorescence-based assays and electrophysiology measurements are developed for discovering ion channel modulators, however, most of the options are time-consuming and costly for high-throughput screening (Zhang et al., 2016). Under the logic that screening of larger libraries could generally lead to higher quality results (Gorgulla et al., 2020), a variety of programs and algorithms have been developed for ultra-high throughput virtual screening. Generally, the ideas for these approaches include optimization in algorithm engineering to enable high-performance computing (Alhossary et al., 2015; Hassan et al., 2017) and the usage of graphics processing units (Santos-Martins et al., 2021; Shidi et al., 2022), the development of highly-organized workflows (Gorgulla et al., 2020), the application of deep learning which trained QSAR models on docking scores of subsets of the library (Gentile et al., 2020), and the improvement in searching efficiency in a fragment-based manner with the idea partial similar with dynamic programming (Sadybekov et al., 2022). These methods have pushed the limit of virtual screening throughput to libraries with more than tens of billion compounds, and may potentially help the drug discovery of various target classes including ion channels.

## Computational Approaches for Functional Analysis of Ion Channels

Recent advances in structural biology such as high-resolution cryo-EM have enabled the in-depth study of the functions and mechanisms of ion channels at the molecular scale. However, protein dynamics is essential for the functions of ion channels, which is difficult to infer from static structures. Therefore, computational approaches have been widely applied to explore the dynamic responses and microscopic interactions of macromolecules (Figure 3).

### Simulation of Ion Conductance

Since the first ion channel structure was published in 1998 by Doyle et al. in their study of the KcsA channel (Doyle et al., 1998), scientists have been using molecular dynamics (MD) simulations to study the interactions between ion channels and their solvent environment. In 2000, Berneche et al. simulated the KcsA K^+^ channel in dipalmitoylphosphatidylcholine (DPPC) phospholipid bilayer with KCl aqueous salt solution (Bernèche and Roux, 2000). Their work showed that the translocation of K^+^ ion along the ion conductance pathway is facilitated by the structural movements of the extracellular residues Glu 71, Asp80, Arg89, and the selectivity filter residues Val76, Gly77. The dynamics of water and K^+^ ions were reported in several simulation studies, where a single file of potassium with water molecules interspersed in between passes along the channel pore (Guidoni et al., 2000; Shrivastava and Sansom, 2000). The observed interactions with the fluctuating selectivity filter laid the foundation for understanding the basis for K^+^ selectivity over Na^+^. Later, Shrivastava et al. showed that K^+^ ions are preferentially coordinated by eight carbonyl oxygens of the filter (Shrivastava et al., 2002), which mimics the salvation shell of hydrated K^+^, while the smaller Na^+^ ion interacts with four carbonyl organs and two water molecules, which exerts larger distortion on the selectivity filter. Further simulation studies revealed that to accommodate the smaller ionic radius of Na^+^ and provide the expected coordination, the dynamic carbonyl groups need to adapt to a “collapsed” state, which builds up unfavorable strain energy. The local interaction mediated by the filter residues and coordinating waters provides a molecular mechanism for ion selectivity in KcsA (Noskov et al., 2004; Noskov and Roux, 2006; Bostick and Brooks, 2007; Noskov and Roux, 2007). After the initial surge of both experimental and simulation studies of the KcsA channel with the availability of its structural information, research has been continuously growing in studying the structures and ion conductance of potassium channels. Long before the crystal structure of the inward rectifier potassium channel Kir6.2 was solved, MD simulation studies had already been conducted on its homology model, which represents another field of computational applications in pursuing structure-based approaches to research (John and Sali, 2003). Capener et al. showed that the Kir6.2 channel model has a similar ion passing motion with KcsA (Capener et al., 2000).

Despite the success of these early studies, a complete permeation event was not described until Khalili-Araghi et al., who applied an electric field of 1 V across the membrane bilayer for the open state of K_v_1.2 channel during 25 ns of MD simulations, and observed permeation events that are consistent with previous studies, where K^+^ ions pass the channel pore through the water-mediated knock-on mechanism (Khalili-Araghi et al., 2006). With increasing computation power, more detailed studies of the ion conductance mechanisms have been carried out. In 2010, Jensen et al. performed simulations on K_v_1.2 with the voltage ranging between −180 and 180 mV over a total of ∼30 μs(Jensen et al., 2010). In this work, not only do they show that the rate-limiting step of K^+^ conductance is the formation of the knock-on intermediate, where two ions form direct contact but also revealed a then-novel gating mechanism that at reverse or zero voltage, the channel undergoes a dewetting transition into the intrinsically more stable closed state.

In addition to the direct application of electric field across the membrane, computational electrophysiology has also been used to study ion permeation. This approach uses a double membrane to separate the system into two isolated compartments, and different ion concentrations are created in each compartment to directly form an electrochemical gradient across the membrane. Köpfer et al. used this method to study the K^+^ conductance of the archaeal MthK channel from *Methanobacterium thermoautotrophicum* and the eukaryotic K_v_1.2-K_v_2.1 chimeric channel in the physiological voltage range (Köpfer et al., 2014). Their results suggest that direct ion-ion contacts instead of co-translocation of alternating ion and water is the key to highly efficient K^+^ conductance. Several subsequent studies reported this direct knock-on mechanism for different potassium channels under a variety of simulation conditions (Schewe et al., 2016; Kopec et al., 2018; Kopec et al., 2019; Lolicato et al., 2020). These studies proposed that strong electrostatic repulsion between ions is the main driving force for the fasting permeation of K^+^ ions, and the higher energetic penalty for complete desolvation of Na^+^ over K^+^ contributes to the ion selectivity. The two permeation mechanisms are still under debate. Both experimental and computational research has been providing various support for either of the mechanisms. It cannot be excluded that a mixture of these mechanisms exists depending on the channel and its physiological conditions.

In contrast to K^+^ channels, the studies of ion conductance of sodium selective channels have been hindered by relatively fewer structural information. The publication of the prokaryotic voltage-gated sodium channel Na_v_Ab from *Arcobacter butzleri* with an activated voltage sensor and a closed pore first provided a structural basis for investigations of the selectivity and transport of Na^+^ ions (Payandeh et al., 2011). A notable difference between K_v_ and Na_v_ channels lies in the selectivity filter. The backbone carbonyl oxygens of the K_v_ channel selectivity filter loop form the cation binding sites to coordinate and pass on K^+^ ions. However, Na_v_ channel selectivity filters are lined by the side chains, which contribute to the relatively wider and more diversely shaped ion pathways in Na_v_ channels. This difference puzzled scientists as to how the wider filter provides selectivity for the ion with a smaller atomic radius and prevents K^+^ and Ca^2+^ to permeate. Corry and Thomas (Corry and Thomas, 2012) calculated the energetics of ion permeation in Na_v_Ab via MD simulations, and showed that a plane formed by four of the filter lining glutamate residues prohibits K^+^ to be rightly coordinated with water molecule bridging between the cation and the carboxylate groups. Similar binding geometry of partially-hydrated Na^+^ has also been reported in the open state sodium channel from the marine bacterium *Magnetococcus* sp. (Na_v_Ms) (McCusker et al., 2012), showing that the glutamate side chains form the high field strength ion binding site, which has a lower permeation barrier for Na^+^ over K^+^ (Ulmschneider et al., 2013). However, the timescale of these studies hampered the observation of concerted movement between ions and proteins. Simulations on the microsecond scale showed that the selectivity filter fluctuates in a coordinated way with ion translocation (Chakrabarti et al., 2013; Boiteux et al., 2014; Ke et al., 2014; Furini and Domene, 2018). With the ion moving across the narrowest part of the pore, the GLU side chains break the symmetric arrangement to coordinate multiple ions to facilitate the passage of the ion into the cavity (Chakrabarti et al., 2013; Boiteux et al., 2014; Guardiani et al., 2017; Callahan and Roux, 2018; Chen et al., 2021). Although simulation studies of bacterial Na_v_ channels have provided valuable insights into the selectivity and ion conductance of Na_v_ channels, structures of eukaryotic Na_v_ channels were not available until 2017 (Shen et al., 2017; Yan et al., 2017; Pan et al., 2018; Shen et al., 2018; Pan et al., 2019; Shen et al., 2019; Jiang et al., 2020). Instead of the homo-tetrameric configuration of the bacterial Na_v_ channels, the four domains of eukaryotic Na_v_ channels are formed by a single polypeptide chain, which gives rise to an asymmetrical pore structure. Compared to the EEEE motif of the prokaryotic SF, eukaryotic SF is constituted by aspartic acid, glutamic acid, lysine, and alanine (DEKA), each from one channel domain. Simulation studies suggested that despite this striking structural difference, the asymmetrical SF functions similarly to the symmetrical prokaryotic counterpart. The ASP and GLU residues coordinate the Na^+^ ion, and the protonated LYS has been proposed to act as another cation in the knock-on or pass-by mechanism (Zhang J. et al., 2018; Flood et al., 2018).

### Analysis of Functional States

Ion channels control the flux of ions by changing between their active and inactive states, which relies on changes between different conformations. Such transitions have proven to be difficult to capture by experimental methods, and even when structures of different states are available, the process of induction and transformation of conformational changes is still hard to explain with the static pictures, which might be important for rational drug design. As a result, computational techniques, especially MD simulations, become useful in studying different function states of ion channels.

As an example, Wen and Zheng performed extensive simulations on the wild-type closed state and the constitutively active mutant of the heat-sensitive cation channel TRPV1 at different temperatures (Wen and Zheng, 2018). They observed a range of hydrogen-bonding network rearrangement between domains in the WT simulations, which are consistent with previous mutational studies, providing a mechanistic explanation of the transition between a closed to pre-open state. Simulations of the gain-of-function mutant complete the process from pre-open to an open state. Similar propagation of movements in the S2-S3 and S4-S5 linkers were observed, suggesting that the dynamic motions in these domains are the key to the channel opening. To study the gating process, Guardiani et al. used targeted molecular dynamics to simulate the transition between closed and open states of the calcium release-activated calcium channel (CRAC) (Guardiani et al., 2021). They reported that upon binding of the activator protein STIM1, a propagation of conformation change occurs from the TM4 to TM1 helices. The extension of TM4 pulls TM3 outward, which in turn moves the lower part of TM1 backward and opens the hydrophobic region of the pore. This result is consistent with patch-clamp experiments on a series of mutants, which showed that disruption of the TM11-TM3 interactions reduces Ca^2+^ influx (Liu et al., 2019).

For mechanosensitive ion channel TREK-2, Aryal et al. simulated changes in membrane tension by changing the area per lipid, and thus studied the effect of lateral pressures on the conformation of the channel (Aryal et al., 2017). They observed that increase in membrane tension induced a transition from the downstate to the upstate. The expansion of the bilayer increases the cross-sectional area of the lower half of the protein, while the upper half remains unchanged to maintain the integrity of the selectivity filter. Interestingly, such membrane stretch does not induce a conformational change in the homologous non-mechanosensitive TWIK-1 channel, indicating that the membrane tension-dependent conformational change is specific to mechanosensitive K2P channels. NOMPC is also a mechanosensitive ion channel, it has, however, been shown to be activated by compression of the intracellular ankyrin repeat domain on the normal plane of the membrane instead of a stretch along the bilayer. In MD simulations, Wang et al. observed that when a pushing force is applied, the TRP domain undergoes an upward movement and clockwise rotation, which induced a rotation in the S6 helices leading to the opening of the channel (Wang et al., 2021). They validated this using patch-clamp experiments and showed that clear electrical signals could be detected when a compressing force was applied, which were abolished with the addition of a NOMPC blocker. Yet, a pulling force was not able to induce a clear NOMPC-dependent current. Another well-known mechanosensitive channel Piezo1 has also attracted a large amount of research attentions. Due to the local bilayer convex curvature imposed by the bowl-like shape of Piezo1, it has been proposed that as membrane tension increases, the protein flattens, which leads to opening of the channel (Guo and MacKinnon, 2017). Since the membrane is a crowded environment, any effects of alteration of the membrane are likely to propagate beyond a single channel. Jiang et al. used a hyperbolic tangent model to study the membrane topology and channel opening as a result of overlapping neighboring Piezo1 membrane footprints. Together with atomistic MD simulations, they found that this overlap decreased bilayer curvature, creating a tension-free opening of Piezo1 (Jiang W. et al., 2021).

Not only has computational methods showed their broad applicability in studying single protein properties, but they also have been used to investigate interactions between channels and their auxiliary proteins. Catte et al. simulated the human K_v_4.3 channel in complex with one of its auxiliary β-subunits, K_v_ channel-interacting protein 1 (KChIP1), and detailed the structural and energetic changes of the complex upon mutations in the interfaces of the complex (Catte et al., 2019). By combining computational protein-protein docking, MD simulations, and electrophysiology, Kuenze and co-workers showed that the S1, S4, S5 helices of voltage-gated KCNQ1 potassium channel interacts with a three-amino-acid motif (F57-T58-L59) in the auxiliary protein KCNE1, which modulates the channel gating through an allosteric network with S5-S6 pore helices (Kuenze et al., 2020). With more structures of channel-auxiliary protein complexes available, such as the sodium leak channel NALCN in complex with auxiliary subunit FAM155A (Xie et al., 2020) or the AMPA-subtype ionotropic glutamate receptor with the auxiliary subunit γ2, more computational investigations can be conducted to reveal the underlying mechanistic details of such interactions (Yelshanskaya et al., 2022).

### Identification of Binding Site and Allosteric Site

Identifying potential binding sites has become a key step in rational drug discovery. Computational methods for identifying protein-ligand binding sites can be roughly classified into sequence-based and structure-based methods. The main assumption for sequence-based approaches is that functionally important binding sites are highly conserved for proteins to maintain their functionalities. Therefore, these methods scan protein sequences for conserved residues as potential binding sites (Capra and Singh, 2007). However, the lack of spatial and physicochemical information in such predictions limits the ability of these methods in providing reliable binding sites. Consequently, structure-based methods have become the mainstream methods for predicting potential binding sites with the advances in obtaining reliable structures both experimentally and computationally. One of the most common approaches in this category is searching for cavities on protein surfaces. Since such methods were pioneered in the 1990s (Levitt and Banaszak, 1992; Laskowski, 1995), developments in this field have not only incorporated both structural and conservation information (Huang and Schroeder, 2006; Capra et al., 2009), but also allowed predictions regarding properties that are highly relevant to drug discovery such as binding affinities (Laurie and Jackson, 2005; Ngan et al., 2012; Liu et al., 2020) and druggability (Le Guilloux et al., 2009; Volkamer et al., 2012; Yuan et al., 2013; Hussein et al., 2015). Methods that infer binding sites from structurally similar template proteins have also been developed (Brylinski and Skolnick, 2008; Wass et al., 2010; Roy and Zhang, 2012; Yang J. et al., 2013). They take advantage of the quick accumulation of protein structures, and provide relatively reliable binding sites for proteins with templates of high structural similarities. In recent years, artificial intelligence methods have also been applied in the prediction of protein-ligand binding sites, which learned the structural or interaction patterns of the binding sites using schemes such as 3D convolutional neural networks (Jiménez et al., 2017) or grid-based approaches (Pu et al., 2019), and may provide satisfactory results when compared with traditional structure-based methods (Capra et al., 2009; Le Guilloux et al., 2009). Additionally, to consider the dynamic nature of ligand binding, MD simulations are also frequently used besides or together with static pocket detection algorithms (Schmidtke et al., 2011; Ung et al., 2016; Chen et al., 2019).

These methods have been widely applied to investigate ligand binding in ion channels. To validate their deep learning-based binding site detection algorithm BiteNet, Igor and Popov applied it to the ATP-gated cation channel P2X3 (Kozlovskii and Popov, 2020). They were able to successfully identify the binding sites for the endogenous agonist ATP and the antagonist AF-219. It is worth noting that such success depends on the protein conformational state. The difference between the agonist-bound and the antagonist-bound conformations prevents the correct prediction of the agonist binding site in the antagonist-bound conformation and vice versa. This is a common limitation of pocket prediction methods using static structures. Nguyen et al. constructed a homology model of human Na_v_1.5 based on electric eel Na_v_1.4 (Yan et al., 2017), and used molecular docking to identify binding locations of antiarrhythmic and local anesthetic drugs, lidocaine, flecainide, and ranolazine (Nguyen et al., 2019). Starting from five different initial locations, they were able to locate binding hot spots of each drug, which are consistent with regions suggested by experimental data. Further multi-microsecond MD simulations revealed detailed mechanistic insights into the dynamic passage and binding of the ligands. Faulkner et al. performed atomistic MD simulations to probe the binding sites of the opioid analgesic fentanyl on the GLIC channel (Faulkner et al., 2019). Fentanyl molecules were placed in a bulk solution to allow free exploration of potential binding sites on the protein, and binding affinities of each site were assessed by molecular mechanics Poisson−Boltzmann surface area (MM/PBSA) calculations (Miller et al., 2012). The newly identified stable binding sites in GLIC are different from previously observed sites for other general anesthetics. They also reported that the binding of fentanyl to one of these novel sites leads to conformational changes, which results in the formation of a hydrophobic gate inhibiting ion conductance through the channel.

Targeting allosteric binding sites of ion channels has aroused more and more attention in recent years considering that the emblematic pore-blocking strategy that targets the highly conserved region might suffer from poor subtype specificity (Marzian et al., 2013). The allosteric effect refers to the phenomenon that the modulation of a binding site (named as allosteric site) differed from the classic active site (referred to as the orthosteric site) resulting in the functional change of the protein. A variety of allosteric sites and the corresponding modulators have been discovered in the field of ion channels, according to that organized in the Allosteric Database (Huang et al., 2011; Huang et al., 2014). Using the comprehensive alanine-scanning mutagenesis, patch-clamp electrophysiological recordings as well as molecular docking and molecular dynamic simulation approaches, Decher et al. showed that the K_v_1 inhibitor Psora-4 could bind to another less conserved site of the channel in addition to the central pore cavity, which demonstrated a new allosteric site of the K_v_1.x channels and thus provided the molecular basis for the development of novel selective voltage-gated channel inhibitors (Marzian et al., 2013). Under the similar strategies, with TKDC, an inhibitor of the TREK subfamily, as the chemical probe, Yang et al. conducted a study combining molecular simulation, mutagenesis, and electrophysiology, and revealed an allosteric site of the two-pore domain potassium (K2P) channel that located in the extracellular cap of the channel (Luo et al., 2017). Accordingly, virtual screening was performed and a series of new inhibitors were identified (Luo et al., 2017). With the combination of molecular dynamics and oocyte electrophysiology studies, Lindahl et al. revealed the special motion and modulation with the modification of a transmembrane binding site within each subunit of the GLIC channel, which thus supports the multisite model of transmembrane allosteric modulation of the channel (Heusser et al., 2018). Using microseconds long atomistic MD simulations, Botello-Smith et al. identified the binding site of the allosteric agonist Yoda1 of Piezo1 (Botello-Smith et al., 2019). Their study indicated that Yoda1 binds at a site approximately 40 A aways from the channel pore, and modulates the channel activity by facilitating force-induced motions of the bound subunit. Similar research was also conducted in various ion channel systems by different groups, including the voltage-gated KCNQ1 potassium channel (Kuenze et al., 2020) and the large-conductance mechanosensitive channel MscL (Kapsalis et al., 2019).

The prediction of potential allosteric sites might also be empowered with the combination of machine learning or deep learning approaches and molecular simulation strategies. The general idea of these algorithms is to analyze the correlation between the orthogonal site and the allosteric site. The prediction could be conducted based on either a single protein structure or combined with trajectories from molecular simulation. In the former case, the correlation might be analyzed based on perturbation exerted on the orthogonal sites using a network model including an elastic network model, anisotropic network model, and graph-based model (Amor et al., 2016; Dokholyan, 2016; Zhang and Nussinov, 2019; Wang J. et al., 2020; Huang et al., 2021; Mersmann et al., 2021; Zheng, 2021; Wu et al., 2022). In the latter case, as described in the cases mentioned above, the results of the simulation-based approaches rely heavily on sampling efficiency. Recently, the reinforced dynamics scheme was reported by Zhang and Wang et al. by constructing the free energy surface with deep neural network models, which turn out to be highly efficient in sampling high-dimensional free energy landscapes (Zhang L. et al., 2018; Wang et al., 2022), and thus might provide new insights into the allosteric modulation of ion channels based on the comprehensive exploration of the protein structural dynamics. Additionally, the evolutionary-based approach could predict the allosteric communication in proteins as well by analyzing the evolutionarily conserved networks of residues (Suel et al., 2003). Case studies also showed that the evolutionary insights could contribute to the understanding of allosteric regulation of the TRP Ion channels (Hilton et al., 2019). However, an in-depth discussion of the principle of the algorithms is beyond the scope of this paper. Details of the algorithms are referred to in the previous literature (Dokholyan, 2016; Liu and Nussinov, 2016; Zhang and Nussinov, 2019).

## Conclusion

Ion channels have long been concerned as medically important drug targets. Increasing the ligand screening throughput and ligand binding selectivity are the main challenges in the search for highly potent ion-channel modulators. As the potential key to meeting these challenges, the practice of rational drug design has long been expected.

Resolving the molecular structures of ion channels is getting easier owing to the rapid development of single-particle cryo-EM. Also, the impressive progress in protein structure prediction is bringing fundamental changes to structural biology. Although accurate predictions of multimeric structures, protein-protein interactions and multiple conformations remain challenging, we are already seeing progress in these directions such as the structural prediction of core eukaryotic protein complexes (Humphreys et al., 2021), heterodimeric protein complexes (Bryant et al., 2022) and alternative conformations for receptors and transporters (Del Alamo et al., 2022). Innovations and applications of protein folding algorithms have been accelerated through open-sourcing AlphaFold2-comparable high-performance training code^3^, and online tools such as ColabFold (Mirdita et al., 2022) and Uni-Fold^4^. It is expected that protein folding algorithms will soon be used to study ion channel conformational states and channel-modulator interactions, even for new channel discoveries. Results from the exploration in the drug discovery era driven by occasions and classic synthetic chemistry enabled reliable computational models through ligand-based approaches. More abundant and higher quality ion channel structures (obtained either experimentally or computationally), as well as accurate binding affinity predictions and ultra-high-throughput virtual screening, have pushed forward structure-based rational drug design. Besides techniques based on static protein structures, simulation and machine-learning-based methods also allow us to explore the dynamic nature of ion channels and direct the rational drug design process. Various computational studies have revealed the molecular mechanisms of channel functions and potential ligand-binding sites, especially allosteric binding sites. With help from these computational methods, we expect the implementation of a more effective drug discovery paradigm in the coming future (de Oliveira et al., 2021; Lees et al., 2021; Robertson et al., 2022).

## Footnotes

1
https://www.rbvi.ucsf.edu/chimerax/data/stanford-sep2021/alphafold.html

2
https://phenix-online.org/documentation/reference/alphafold_in_colab.html

3
https://github.com/dptech-corp/Uni-Fold

4 Hermite platform: https://hermite.dp.tech
